# Development of Targeted Mass Spectrometry-Based Approaches for Quantitation of Proteins Enriched in the Postsynaptic Density (PSD)

**DOI:** 10.3390/proteomes7020012

**Published:** 2019-04-02

**Authors:** Rashaun S. Wilson, Navin Rauniyar, Fumika Sakaue, TuKiet T. Lam, Kenneth R. Williams, Angus C. Nairn

**Affiliations:** 1 Yale/NIDA Neuroproteomics Center, New Haven, CT 06511, USA; rashaun.wilson@yale.edu (R.S.W.); tukiet.lam@yale.edu (T.T.L.); kenneth.williams@yale.edu (K.R.W.); 2 W.M Keck Biotechnology Resource Laboratory, Yale University School of Medicine, New Haven, CT 06511, USA; 3 Molecular Biophysics and Biochemistry, Yale University School of Medicine, New Haven, CT 06511, USA; 4 Tanvex BioPharma Inc., San Diego, CA 92121, USA; navin.rauniyar@tanvex.com; 5 Department of Neurology and Neurological Science, Tokyo Medical and Dental University, Tokyo 113-8519, Japan; sakaue.nuro@tmd.ac.jp; 6 Department of Psychiatry, Yale School of Medicine, Connecticut Mental Health Center, New Haven, CT 06511, USA

**Keywords:** postsynaptic density, PSD, parallel reaction monitoring, PRM, targeted proteomics, data-independent acquisition, DIA, quantitative mass spectrometry

## Abstract

The postsynaptic density (PSD) is a structural, electron-dense region of excitatory glutamatergic synapses, which is involved in a variety of cellular and signaling processes in neurons. The PSD is comprised of a large network of proteins, many of which have been implicated in a wide variety of neuropsychiatric disorders. Biochemical fractionation combined with mass spectrometry analyses have enabled an in-depth understanding of the protein composition of the PSD. However, the PSD composition may change rapidly in response to stimuli, and robust and reproducible methods to thoroughly quantify changes in protein abundance are warranted. Here, we report on the development of two types of targeted mass spectrometry-based assays for quantitation of PSD-enriched proteins. In total, we quantified 50 PSD proteins in a targeted, parallel reaction monitoring (PRM) assay using heavy-labeled, synthetic internal peptide standards and identified and quantified over 2100 proteins through a pre-determined spectral library using a data-independent acquisition (DIA) approach in PSD fractions isolated from mouse cortical brain tissue.

## 1. Introduction

The postsynaptic density (PSD) is an electron-dense region of excitatory glutamatergic synapses located just beneath the postsynaptic membrane. The PSD was first discovered by electron microscopy in 1956 [1] and was later found to consist of 30–50 nm-thick, disc-shaped protein structures [2,3]. Within these protein structures are several classes of protein families, many of which are involved in processes such as scaffolding and signal transduction. Each of these families are organized in two different structural layers of the PSD: the core and the pallium [4]. The core is the structural layer located near the postsynaptic membrane, while the pallium is positioned beneath the core and is thought to be more labile. 

**Figure 1:**
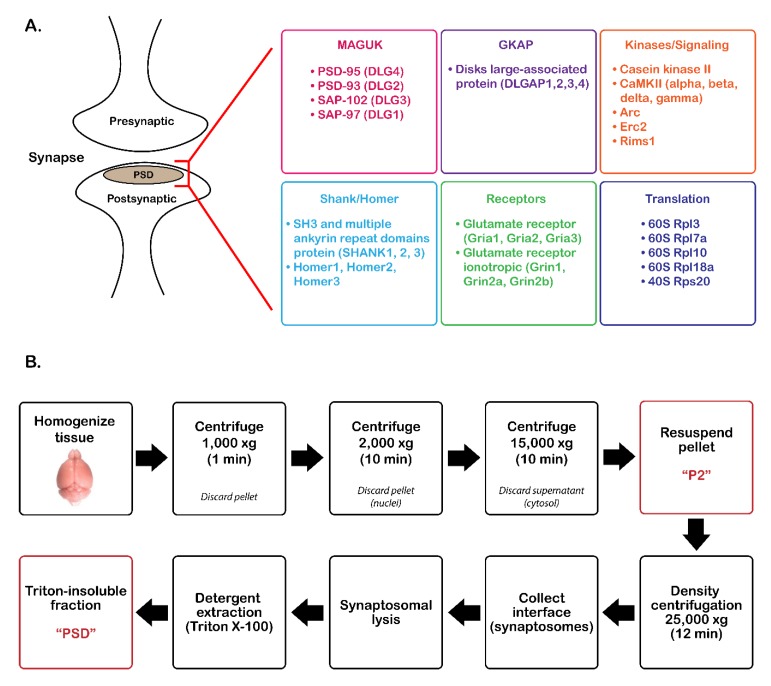
Overview of postsynaptic density (PSD) protein enrichment from mouse cortical tissue. (**A**) List of groups of commonly identified proteins in the PSD. (**B**) Steps for PSD enrichment starting from tissue homogenization to Triton X-100 precipitation. MAGUK, membrane-associated guanylate kinases, GKAP, guanylate kinase-associated proteins, DLGAP, disks large-associated proteins.

One group of proteins that has previously been identified in the PSD core is the membrane-associated guanylate kinases (MAGUKs) [5,6,7,8] (Figure 1A). These proteins are comprised of three main domains including the PDZ, SH3, and guanylate kinase (GK) domains [5,9,10]. One of the most abundant proteins within the MAGUK family is PSD-95 (also known as DLG4 or SAP90) [7,8], which is involved in structural maintenance and signaling through interactions with integral membrane proteins and receptors, protein complexes, and other structural proteins within the PSD [10,11,12]. In addition to PSD-95, the MAGUK family includes PSD-93 (DLG2), SAP-102 (DLG3), and SAP-97 (DLG1).

Guanylate kinase-associated proteins (GKAPs) are another class of proteins found in the PSD core. This family was first isolated by Kim et al. [13] and found to directly bind to the GK domains of MAGUKs through co-immunoprecipitation assays and immunohistochemistry. GKAPs are often referred to as disks large-associated proteins (DLGAPs, also referred to as SAPAPs), which include four different isoforms designated DLGAP1,2,3, and 4 (Figure 1A). These isoforms enable the formation of protein complexes with MAGUKs and proteins found in the pallial layer of the PSD.

An additional protein family present in the PSD pallium and associated with these complexes is the SH3 and multiple ankyrin repeat domain protein (Shank) family. As their name suggests, these proteins contain an SH3 domain, as well as ankyrin repeats, a PDZ domain, a proline-rich domain, and a SAM domain. These proteins were first identified by Naisbitt et al. [14], who demonstrated that the C-terminal region of GKAP binds to the PDZ domain of Shank. There are three Shank isoforms (Shank1,2,3) that are capable of binding to both MAGUKs and GKAPs and have been shown to form a PSD-95–SAPAP–SHANK complex [9,13,15]. This complex has been implicated in scaffolding and organization of signaling complexes at glutamatergic synapses [15,16].

The Homer family of proteins is also found in the PSD pallium. The two characteristic structural regions of Homer proteins include an Enabled/vasodilator-stimulated phosphoprotein homology 1 (EVH1) domain [17,18,19,20] and a carboxyl-terminal coiled-coil domain [17,19,21,22,23,24,25]. Homer proteins self-polymerize and interact with Shank proteins, creating a matrix-like structure [4,24,26]. This scaffolding structure is involved in excitatory signal transduction as well as in receptor plasticity [27]. There are three different *Homer* genes (1–3) that are differentially expressed throughout the brain [27].

In addition to structural proteins, protein kinases are an important component of the signaling pathways within the PSD pallium. Ca^2+^/calmodulin-dependent protein kinase II (CaMKII) is a serine-threonine kinase that comprises approximately 1–2% of the total proteome in the cerebral cortex and hippocampus [28]. Studies have shown a marked accumulation of CaMKII at the PSD with increasing levels of neuronal excitation [4,29,30,31,32]. CaMKII has also been implicated in NMDA-dependent long-term potentiation (LTP) [4,33,34] through regulation of its activity by Ca^2+^/calmodulin and autophosphorylation.

There is increasing interest in understanding the functions of proteins in the intricate PSD network because of their potential involvement in a wide variety of neuropsychiatric disorders. For instance, several reports have linked Shank3 to autism spectrum disorder (ASD) [15,35,36]. Specifically, deletion of the Shank3B isoform in mice resulted in an ASD-like, compulsive grooming phenotype, which was more prominent than the other Shank3-associated phenotypes investigated in this report [15]. Another study observed a similar excessive grooming phenotype of a different genetic Shank3B knockout mouse, further implicating Shank3B in ASD-like behavioral disorders [35]. Similarly, mutations in either PSD-95 or SynGAP have been shown to be associated with intellectual disorders and autism [37,38,39]. Furthermore, PSD proteins such as DLG isoforms, DLGAP1, Gria2/3, Grin2a/b, CaMKII, and Homer isoforms have all been implicated in schizophrenia, among many other disorders [40,41,42]. It is apparent from this growing list that studying the organization and function of proteins within the PSD has become an important focus in neuroscience research. Fractionation methods for enriching PSD proteins were developed decades ago [2,43,44,45]; however, because the PSD is not enclosed in a bilayer, it can be challenging to minimize contamination of the PSD fraction with other subcellular proteins [46]. Apart from the enrichment method, the structure of synapses themselves can make analyses difficult. Synapses differ significantly from one another and can change their composition rapidly, making reproducibility and accuracy of the analysis important [47,48,49,50]. Despite these challenges, researchers have made significant efforts to study the proteome of the PSD, particularly through the use of mass spectrometry [27,42,51,52,53,54,55,56,57,58]. Note, however, that many of the PSD fractionation methods use Triton-X100, a detergent that is not compatible with mass spectrometry analysis. Therefore, care must be taken when preparing a PSD fraction to minimize detergent interference.

Mass spectrometry analysis of PSD fractions from mouse and human cortical tissue identified 1556 and 1461 proteins, respectively [53,54]. Interestingly, there was a 70% overlap of proteins in the mouse and human PSDs. A later study identified 2876 PSD-associated proteins from mouse brain tissue using immunopurification prior to mass spectrometry analysis [57]. Recently, label-free quantitation was performed on 48 PSD samples from 12 human neocortical brain regions, identifying 1213 proteins in total [51]. While these discovery studies have made significant progress identifying PSD proteins, targeted mass spectrometry-based assays are needed to provide the highest possible sensitivity, quantification precision, and accuracy [59]. Our group previously used multiple reaction monitoring (MRM) coupled with stable-isotope peptide standards (SIS) to quantify 112 rat synaptic proteins [58]. Though this assay has made significant improvements in the quantitation of PSD proteins, it lacks high mass accuracy.

Currently, there are two major approaches for targeted, high-mass-accuracy quantitative mass spectrometry. The first method is data-independent analysis (DIA), which was first proposed by Venable et al. [60]. DIA uses sequential window acquisition to fragment and quantify all precursor and product ions within a sample [61,62,63,64]. Unlike data-dependent methods, DIA offers high reproducibility and quantitation, while maintaining sensitivity at higher levels of multiplexing [65]. One study has already demonstrated the use of DIA analysis on fractionated PSD samples from mouse hippocampal tissue, which resulted in the identification of 2102 protein groups in the PSD fractions [66]. The second approach is a more targeted method called parallel reaction monitoring (PRM). Like MRM/selected reaction monitoring (SRM) methods, PRM offers similar accuracy and reproducibility; however, it provides a wider dynamic range and improved selectivity [67,68,69].

Given the advantages of these targeted methods, we developed new DIA and PRM assays to quantify PSD proteins. For the PRM assay, heavy labeled peptides were synthesized and used as internal standards for accurate protein quantitation. Two different mouse datasets were used to evaluate the performance of these methods: PSD-enriched fractions versus pre-fractionation, and wild-type (WT) versus Shank3B knockout (KO) PSD fractions. These assays enabled accurate quantitation of PSD proteins and provide promising tools for future PSD proteomics studies.

## 2. Materials and Methods

### 2.1. Tissue Collection

Wild-type and Shank3B mouse cortical tissue was isolated and frozen on dry ice prior to protein extraction. 

### 2.2. PSD Enrichment

PSD isolation was adapted from previously described methods [2]. In brief, mouse cortical brain tissue (~100 mg/sample) was homogenized on ice in 1 mL Buffer A (5 mM HEPES, 10% sucrose (w/v), 1X cOmplete, Mini, EDTA-free protease inhibitor cocktail (Roche Diagnostics GmbH, Mannheim, Germany)) using a rotary homogenizer (Glas-Col, LLC, Terre Haute, IN, motor size: 4.38"w × 4.38"d × 5.50"h) for 10 strokes at a speed of 40. The lysate was spun in a tabletop centrifuge at 1000× g for 1 min at 4 °C (Figure 1) to remove cellular debris. The supernatant was transferred to a new Eppendorf tube and centrifuged at 2000× g for 10 min at 4 °C to remove the nuclei. The supernatant was transferred to a new tube and centrifuged at 15,000× g for 10 min at 4 °C. The supernatant (cytosolic fraction) was discarded, and the pellet, which contains synaptosome/synaptoneurosomes, was resuspended in three volumes of Buffer A (P2 fraction). The sample was applied to the top of a Percoll gradient (3-23% in Buffer A; GE Healthcare, Chicago, IL, USA) and centrifuged in an Optima MAX-XP Ultracentrifuge (Beckman Coulter, Brea, CA, USA) at 25,000× g (MLA-55 rotor) for 12 min at 4 °C. The interface containing synaptosomes was collected between 15–23% Percoll. The synaptosomal fraction was subjected to hypotonic lysis by suspending in three volumes of Buffer B (5 mM HEPES, 1 mM DTT, 1X cOmplete, Mini, EDTA-free protease inhibitor cocktail) for 30 min on ice. The lysate was centrifuged at 25,000× g (MLA-55 rotor) for 30 min at 4 °C. The pellet was resuspended in 2 mL Buffer C (0.75% Triton X-100 in Buffer A) and incubated on ice for 15 min (detergent extraction). The sample was centrifuged at 63,000× g (MLA-55 rotor) for 30 min at 4 °C. The supernatant (detergent-soluble fraction) was removed, and the pellet (detergent-insoluble PSD fraction) was washed three times with 1 mL phosphate-buffered saline (PBS). The pellet was resuspended in 8 M urea, 400 mM ammonium bicarbonate, and stored at −20 °C.

### 2.3. Immunoblot Analysis

Proteins (10 µg) were resolved using 4–20% gradient gels (Invitrogen, Carlsbad, CA, USA) and then transferred to PVDF membranes that then were blocked for 1 h with blocking buffer (LI-COR Biosciences, Lincoln, NE). Primary antibodies were diluted 1:5000 in blocking buffer prior to membrane incubation overnight at 4 °C. Blots were washed four times with phosphate buffered saline with Tween 20 (PBST) (0.05% v/v) and incubated with IRDye secondary antibody (LI-COR Biosciences) (1:10,000 dilution in PBST (0.5% v/v)). Blots were imaged using a LI-COR Odyssey Imaging System (LI-COR Biosciences). Immunoblot quantitation was performed using Image Studio Software v. 5.2.5 (LI-COR Biosciences).

### 2.4. Sample Preparation for LC–MS/MS

PSD protein fractions were quantified using the Bradford method [70]. Proteins (50 µg) were placed into an Eppendorf tube, and the volume was brought to 100 µL with 8 M Urea, 400 mM ammonium bicarbonate. Proteins were reduced with 10 µL of 45 mM DTT and incubated at 37 °C for 30 min. They were then alkylated with 10 µL of 100 mM iodoacetamide (IAM) and incubated in the dark at room temperature for 30 min. After diluting with water to bring urea concentration to 2 M, sequencing-grade trypsin (Promega, Madison, WI, USA) was added at a weight ratio of 1:20 (trypsin/protein), and the fractions were incubated at 37 °C for 16 h. The samples were desalted using C18 spin columns (The Nest Group, Inc., Southborough, MA, USA) and dried in a rotary evaporator. The samples were resuspended in 0.2% trifluoroacetic acid (TFA) and 2% acetonitrile (ACN) in water prior to LC–MS/MS analysis.

### 2.5. Parallel Reaction Monitoring (PRM) Method Development

#### 2.5.1. Peptide Design and Synthesis

A list of peptides was generated from previous DDA and DIA analyses of PSD fractions isolated from rat brain tissue. Candidate PRM peptides were selected from this list on the basis of the following criteria: 1) the peptide must be 8–30 amino acids in length and have the same sequence in both mice and rats, 2) the peptide must contain a minimal number of modifiable residues (Met, Cys, Ser, Thr, Tyr), and 3) the peptide must have a minimal number of flanking Arg and Lys residues to avoid miscleavage events. Stable-isotope-labeled (SIL) peptides were synthesized as SpikeTides TQL PLUS peptides and then robotically pooled by JPT Peptide Technologies, GmbH (Berlin, Germany). 

#### 2.5.2. SIL Peptide Dilution Series (Neat)

A six-point, two-fold dilution series was performed from 75–3000 fmol per peptide. The peptides were reduced with 45 mM DTT and incubated at 37 °C for 30 min. The peptides were alkylated with 100 mM IAM and incubated at room temperature for 30 min in the dark. Sequencing-grade trypsin (Promega, Madison, WI, USA) was added at a weight ratio of 1:20 (trypsin/protein), and the samples were incubated at 37 °C for 16 h to remove the C-terminal QTag that can be cleaved by tryptic digestion. The samples were desalted using C18 spin columns (The Nest Group, Inc., Southborough, MA, USA) and dried in a rotary evaporator. The samples were resuspended in 0.2% TFA and 2% ACN in water. Each dilution was injected in technical triplicates, resulting in 25, 50, 100, 250, 500, and 1,000 fmol of each peptide being injected on the column. Results (peak area intensities, dot products (dotp), mass error, and retention times) from this analysis are displayed in Table S1. 

#### 2.5.3. SIL Peptide Dilution Series in Fixed Biological Peptide Matrix

A six-point, two-fold dilution series was performed from 75–3000 fmol per peptide in triplicate. Each dilution was added to a fixed amount (10 µg) of three independent biological protein extracts from mouse brain tissue. Each dilution was reduced with 45 mM DTT and incubated at 37 °C for 30 min. The peptides were alkylated with 100 mM IAM and incubated at room temperature for 30 min in the dark. Sequencing-grade trypsin (Promega, Madison, WI, USA) was added at a ratio of 1:20 (trypsin:protein), and the samples were incubated at 37 °C for 16 h. The samples were desalted using C18 spin columns (The Nest Group, Inc., Southborough, MA, USA) and dried in a rotary evaporator. The samples were resuspended in 0.2% TFA and 2% ACN in water. Each dilution was injected in technical triplicates, resulting in 25, 50, 100, 250, 500, and 1000 fmol per SIL peptide and 2–3 µg biological peptide matrix injected on the column. Results (peak area intensities, dotp, mass error, and retention times) from this analysis are displayed in Table S2. Response ratios (heavy vs light peak areas) from this analysis are listed for each peptide in Table S3 along with the corresponding linear performance in Table S4. A linear performance comparison of the SIL peptide in the neat versus fixed matrix analysis series can be found in Table S5.

### 2.6. LC–MS/MS

#### 2.6.1. Data-Independent Acquisition (DIA)

DIA LC–MS/MS was performed using a nanoACQUITY UPLC system (Waters Corporation, Milford, MA, USA) connected to an Orbitrap Fusion Tribrid (ThermoFisher Scientific, San Jose, CA, USA) mass spectrometer. After injection, the samples were loaded into a trapping column (nanoACQUITY UPLC Symmetry C18 Trap column, 180 µm × 20 mm) at a flow rate of 5 µL/min and separated with a C18 column (nanoACQUITY column Peptide BEH C18, 75 µm × 250 mm). The compositions of mobile phases A and B were 0.1% formic acid in water and 0.1% formic acid in ACN, respectively. The peptides were eluted with a gradient extending from 6% to 35% mobile phase B in 90 min and then to 85% mobile phase B in additional 15 min at a flow rate of 300 nL/min and a column temperature of 37 °C. The data were acquired with the mass spectrometer operating in a data-independent mode with an isolation window width of 25 m/z. The full scan was performed in the range of 400–1,000 m/z with “Use Quadrupole Isolation” enabled at an Orbitrap resolution of 120,000 at 200 m/z and automatic gain control (AGC) target value of 4 × 10^5^. Fragment ions from each peptide MS2 were generated in the C-trap with higher-energy collision dissociation (HCD) at a collision energy of 28% and detected in the Orbitrap at a resolution of 60,000.

#### 2.6.2. Parallel Reaction Monitoring (PRM)

PRM LC–MS/MS was performed using a nanoACQUITY UPLC system (Waters Corporation, Milford, MA, USA) connected to an Orbitrap Fusion Tribrid (ThermoFisher Scientific, San Jose, CA, USA) mass spectrometer. After injection, the samples were loaded into a trapping column (nanoACQUITY UPLC Symmetry C18 Trap column, 180 µm × 20 mm) at a flow rate of 5 µL/min and separated with a C18 column (nanoACQUITY column Peptide BEH C18, 75 µm × 250 mm). The compositions of mobile phases A and B were 0.1% formic acid in water and 0.1% formic acid in ACN, respectively. The peptides were eluted with a gradient extending from 6% to 35% mobile phase B in 90 min and then to 85% mobile phase B in additional 15 min at a flow rate of 300 nL/min and a column temperature of 37◦C. The data were acquired with the mass spectrometer operating in targeted mode with a MS^2^ isolation window of 1.6 m/z. The full scan was performed in the range of 350–1,200 m/z with “Use Quadrupole Isolation” enabled at an Orbitrap resolution of 120,000 at 200 m/z and AGC target value of 4 × 10^5^. The MS^2^ scan range was set to 100–2,000 m/z. Fragment ions from each peptide MS^2^ were generated in the C-trap with HCD at a collision energy of 28% and were detected in the Orbitrap at a resolution of 60,000.

### 2.7. Data Analysis

#### 2.7.1. Data-Independent Acquisition (DIA)

DIA spectra were searched against a peptide library generated from DDA spectra using Scaffold DIA software v. 1.1.1 (Proteome Software, Portland, OR, USA). Within Scaffold DIA, raw files were first converted to the mzML format using ProteoWizard v. 3.0.11748. The samples were then aligned by retention time and individually searched against a *Mus musculus* proteome database exported from UniProt with a peptide mass tolerance of 10 ppm and a fragment mass tolerance of 10 ppm. The data acquisition type was set to “Non-Overlapping DIA”, and the maximum missed cleavages was set to 1. Fixed modifications included carbamidomethylation of cysteine residues (+57.02). Peptides with charge states between 2 and 3 and 6–30 amino acids in length were considered for quantitation, and the resulting peptides were filtered by Percolator v. 3.01 at a threshold FDR of 0.01. Peptide quantification was performed by EncyclopeDIA v. 0.6.12 [71], and six of the highest quality fragment ions were selected for quantitation. Proteins containing redundant peptides were grouped to satisfy the principles of parsimony, and proteins were filtered at a threshold of two peptides per protein and an FDR of 1%. Significance was determined using a two-tailed student’s *t*-test. 

#### 2.7.2. Parallel Reaction Monitoring (PRM)

PRM spectra were analyzed by Skyline software v. 4.2.0.19009 (MacCoss Lab, University of Washington) [72]. Three to six transition ion peak area intensities were integrated and summed for each peptide (heavy and light) (See mass list in Table S6). The ratio of light/heavy peak areas was calculated and mean-normalized to obtain a final quantification value for each peptide. Protein quantitation values were then calculated by summation of the peptide quantitative values. Significance was determined using a two-tailed student’s t-test. 

## 3. Results

### 3.1. Validation of PSD Enrichment

A previously optimized enrichment protocol, which requires density centrifugation with a Percoll gradient followed by Triton-X100 precipitation of the PSD fraction (Figure 1B), was used to enrich PSD proteins from four biological WT and Shank3B KO mouse brain tissue and three additional biological replicate WT mouse brain tissue samples. Immunoblot analysis compared protein expression of PSD-95 (PSD marker), GAPDH (cytosolic marker), and prohibitin (mitochondrial marker) in the P2 and PSD fractions isolated from each biological replicate of WT and Shank3B KO tissue (Figure S1A) and in pre-fractionation (PF) (supernatant from Step 2 of Figure 1B) and PSD-enriched (PSD) samples isolated from wild-type tissue (Figure S2A). Immunoblot quantitation revealed that the PSD-enriched fraction displayed a higher ratio of PSD-95/GAPDH expression when compared to the P2 fraction (Figure S1B) or the pre-fractionation samples (Figure S2B) in all biological replicates. From these results, it was apparent that the PSD fraction isolated from all biological replicates was enriched for a PSD marker while also being depleted of cytosolic and mitochondrial contaminants, indicating that these samples were suitable for mass spectrometry-based quantitation of PSD proteins. 

### 3.2. DIA Results Indicated Minor Differences Between WT and Shank3B KO PSD-Enriched Proteins

**Figure 2:**
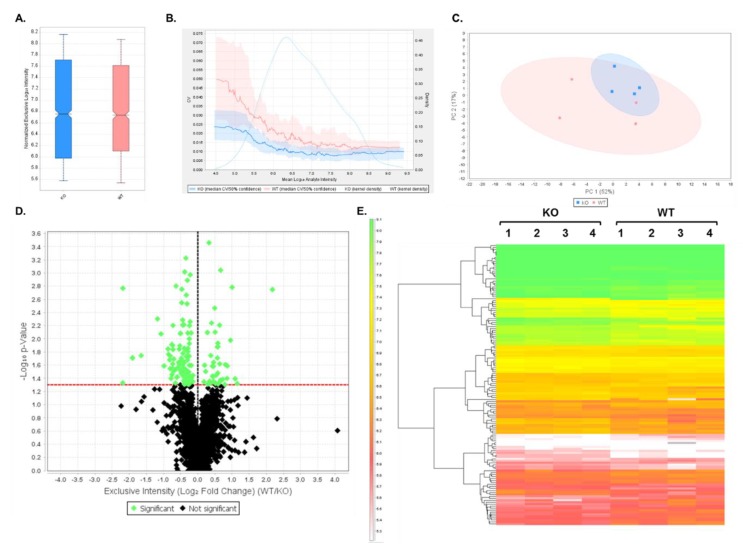
Data-independent analysis (DIA) results comparing wild-type (WT) and Shank3B knockout (KO) samples. (**A**) Box plot displaying quartile, median-normalized log_10_ intensities for each sample group. (**B**) Quantitative CVs chart. The bold lines show the relationship between the mean log_10_ protein intensity and the CV values for WT (pink) and KO (blue) samples. The shaded areas around the plotted lines represent the 50% confidence interval for the CV values. The faint lines indicate the intensity distribution for all proteins within WT (pink) and KO (blue) samples, which were calculated using a Gaussian kernel density estimate. (**C**) Principal Component Analysis (PCA). PCA plot displays the distribution of PC1 and PC2 in WT (pink) and KO (blue) samples. The percentages (%) in each axis represent the explained variance for each Principal Component. (**D**) Volcano plot displaying the log_10_ p-values for each protein as a function of log_2_ fold change (WT/KO) values after performing a t-test. Proteins that are significantly (*p* < 0.05, uncorrected values) changing in expression between the two groups are highlighted in green, while non-significant proteins are shown in black. (**E**) Heatmap of differentially expressed proteins (*p* < 0.05) after t-test statistical analysis. In total, 140 proteins were differentially expressed between WT and KO replicate samples. Hierarchical clustering tree is displayed on the left of the heatmap. The heatmap scale units are in log_10_ intensity.

DIA analysis was first performed on WT and Shank3B KO PSD-enriched fractions to demonstrate the utility of this assay by its ability to detect decreased expression of the Shank3B protein in the KO extracts. Data were analyzed using Scaffold DIA software. Across all samples, a total of 12,699 peptides were identified corresponding to 1862 proteins at two peptides per protein and a 1% protein FDR. The results from this analysis are displayed in Table S7. Between the two samples, the WT and KO fractions displayed similar median intensities of 4.53 × 10^6^ and 4.33 × 10^6^, respectively (Figure 2A) after quartile median normalization. The quantitative CV graph (Figure 2B) indicates that both the WT and KO CV values were below 5% over the entire range of intensities, suggesting low biological variability between samples within each group. In addition, both groups displayed a normal intensity distribution, which was calculated using a Gaussian kernel density estimate. Principal Component Analysis (PCA) was also performed using Scaffold DIA to observe differences between sample groups. These results showed PC1 and PC2 having a 52% and 17% explained variance, respectively, at a 95% confidence interval (Figure 2C). These results indicated a significant overlap of the WT and KO groups when plotting PC1 against PC2, suggesting minor differences between the samples in each group. A two-tailed t-test was then performed between WT and KO samples to determine which proteins had significant differences in expression (*p* < 0.05). A volcano plot was generated to display the log_10_ p-value as a function of the corresponding log_2_ fold change (WT/KO) in expression for all of the identified proteins (Figure 2D). In this plot, the points highlighted in green represent proteins whose expression significantly differed (*p* < 0.05) between WT and KO samples, while the proteins whose expression was not significantly changed are shown in black. In total, the 140 proteins that are listed in Table S8 were found to have significant differences in expression between these two groups. The 140 proteins that had statistically significant differences in expression levels were then displayed in a heatmap, which also shows hierarchical clustering between groups (Figure 2E). 

### 3.3. Expression Profiles from DIA Analysis of Wild-Type and Shank3B KO PSD Fractions Revealed Shank3-Associated Patterns

**Figure 3:**
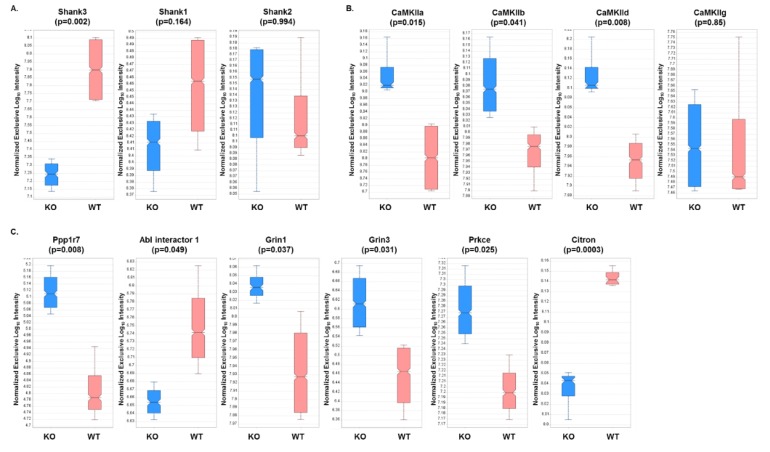
Relative expression levels of several mouse brain proteins based on DIA analyses of WT and KO samples. Expression levels and associated p-values (t-test) are displayed for (**A**) Shank isoforms, (**B**) CaMKII subunits, and (**C**) known Shank3-interacting proteins.

Next, individual expression patterns were examined for some of the proteins identified in the analysis. Not surprisingly, Shank3 displayed a five-fold significant decrease in expression in the KO versus WT fractions, while no significant differences in expression were observed for Shank 1 or 2 (Figure 3A). Since the Shank3 protein has 10 expressed isoforms in mice, it can be expected that partial expression of Shank3 will be present even in the absence of the Shank3B isoform. In addition, three out of four of the CaMKII isoforms displayed a significant increase in expression in KO fractions compared to WT fractions (Figure 3B). This result was particularly interesting, as several CaMKII isoforms have previously been shown to interact with Shank3 [41,73]. In addition, several other known Shank3-interacting proteins were found to have significantly different expression in KO compared to WT fractions (Figure 3C) [56,57]. 

### 3.4. DIA Analyses Indicated Significant Differences in Protein Expression between Pre-Fractionation and PSD-Enriched Samples

**Figure 4:**
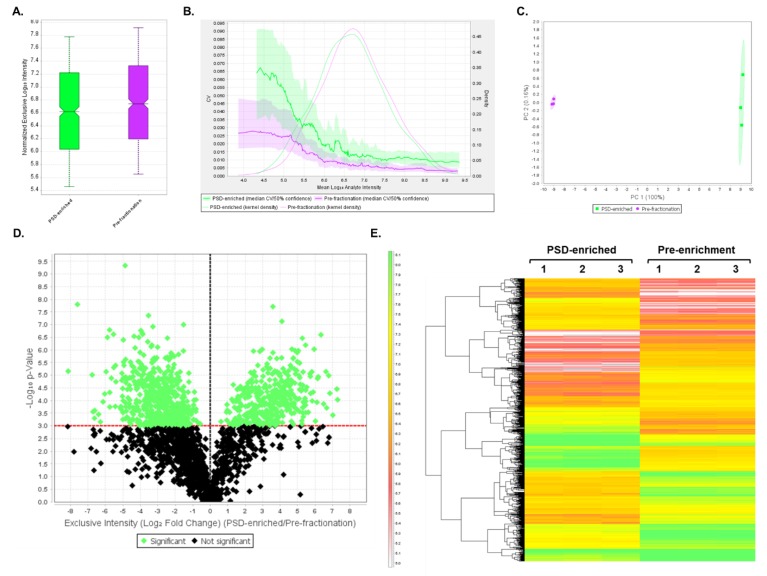
DIA results comparing PSD-enriched and pre-fractionation samples. (**A**) Box plot displaying quartile, median-normalized log_10_ intensities for each sample group. (**B**) Quantitative CVs chart. The bold lines show the relationship between the mean log_10_ protein intensity and the CV values for PSD-enriched (green) and pre-fractionation (purple) samples. The shaded areas around the plotted lines represent the 50% confidence interval for the CV values. The faint lines indicate the intensity distribution for all proteins within PSD-enriched (green) and pre-fractionation (purple) samples, which were calculated using a Gaussian kernel density estimate. (**C**) PCA. PCA plot displays the distribution of PSD-enriched (green) and pre-fractionation (purple) samples between PC1 and PC2. The percentages (%) in each axis represent the explained variance for each Principal Component. (**D**) Volcano plot displaying the log_10_ p-values for each protein as a function of log_2_ fold change (PSD-enriched/Pre-fractionation) values after performing a t-test. Proteins that are significantly (*p* < 0.05, uncorrected values) changing in expression between the two groups are highlighted in green, while proteins whose expression does not significantly differ are shown in black. (**E**) Heatmap of significantly differentially expressed proteins (*p* < 0.05) after t-test statistical analysis. In total, 1721 proteins were differentially expressed between PSD-enriched and pre-fractionation replicate samples. A hierarchical clustering tree is displayed on the left of the heatmap. The heatmap scale units are in log_10_ intensity.

To quantify changes in abundance between proteins present prior to fractionation compared to those in the PSD-enriched fractions, DIA analysis was first performed on three biological samples per group, and the resulting data were analyzed using Scaffold DIA. This experiment demonstrated the utility of the DIA assay to analyze the same set of proteins in both PSD-enriched and unfractionated mouse brain samples. Across all samples, a total of 14,273 peptides were identified corresponding to 2134 proteins at 2 peptides per protein and a 1% protein FDR. Results from this analysis are displayed in Table S9. Between the two samples, the PSD-enriched and pre-fractionation samples displayed median intensities of 4.17 × 10^6^ and 5.50 × 10^6^, respectively (Figure 4A) after quartile median normalization. The quantitative CV graph (Figure 4B) indicated that both pre-fractionation and PSD-enriched CV values were below 5% over the entire range of intensities, suggesting low biological variability between samples within each group. In addition, both groups displayed a normal intensity distribution, which was calculated using a Gaussian kernel density estimate. PCA analysis performed using Scaffold DIA showed PC1 and PC2 having a 92% and 3.9% explained variance, respectively, at a 95% confidence interval (Figure 4C). These results indicated a significant divergence between the PSD-enriched and the pre-fractionation groups when plotting PC1 against PC2. A two-tailed t-test was then performed between PSD-enriched and pre-fractionation samples to determine which proteins had significant differences in expression (*p* < 0.05). A volcano plot was generated to display the log_10_ p-value as a function of the corresponding log_2_ fold change (PSD-enriched/pre-fractionation) for all of the identified proteins (Figure 4D). In this plot, the points highlighted in green represent proteins whose expression significantly differed (*p* < 0.05) between samples, while the proteins whose expression did not significantly differ are shown in black. In total, 1721 proteins, listed in Table S10, were found to have significantly different expression between groups. These proteins with significantly different expression levels were then displayed in a heatmap, which also shows hierarchical clustering between groups (Figure 4E). 

### 3.5. DIA Expression Profiles Displayed Enrichment of PSD Proteins and Depletion of Contaminants in PSD-Enriched Fractions Comparerd to Pre-Fractionation Samples

**Figure 5:**
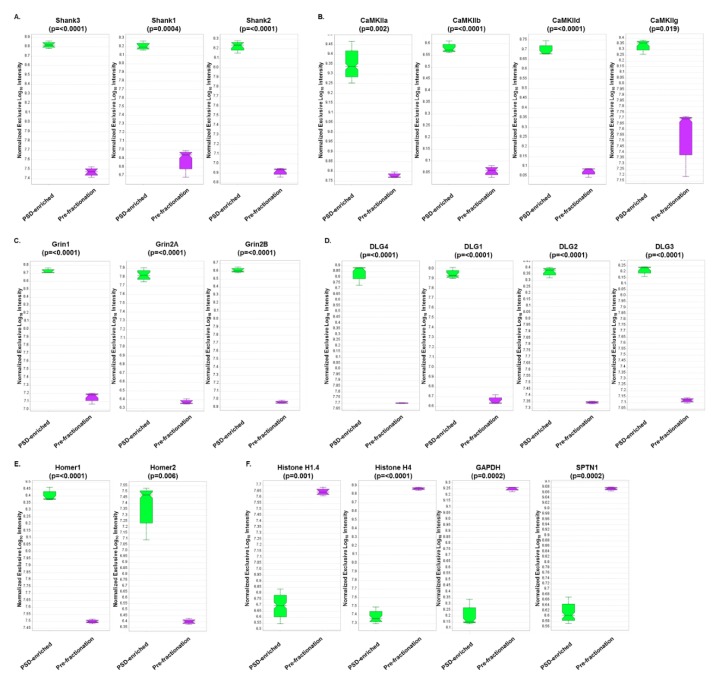
Expression profile results from DIA analysis comparing PSD-enriched and pre-fractionation samples. Expression profiles and associated p-values (t-test) are displayed for (**A**) Shank isoforms, (**B**) CaMKII subunits, (**C**) Glutamate receptors (NMDA), (**D**) Disks-large isoforms, (**E**) Homer isoforms, and (**F**) PSD contaminants.

To quantify the degree of PSD enrichment, expression profiles of PSD protein families were analyzed (Figure 5). Significant increases (*p* < 0.05) in protein expression in PSD fractions compared to pre-fractionation samples were observed for the Shank family (Figure 5A), CaMKII subunits (Figure 5B), ionotropic glutamate receptors (Figure 5C), Disks-large family (Figure 5D), and Homer family (Figure 5E). Conversely, expression patterns of PSD contaminating proteins such as histones (nuclear), GAPDH (cytoplasmic), and alpha spectrin (cytoskeletal) were all significantly decreased in the PSD fractions compared to the pre-fractionation samples (Figure 5E). These results confirmed that the PSD fractions were significantly enriched for known PSD proteins and depleted of other cellular contaminants. Furthermore, this suggests that the DIA assay can be utilized for quantitation of both fractionated and unfractionated brain samples.

### 3.6. Peptide Design for PRM Analysis

The PSD/PRM assay contains 47 proteins that were shown to be from 1.2 to 3.6-fold enriched in the PSD compared to the P2 fraction. In addition, this assay also includes another PSD protein, Csnk2a1 (Casein Kinase 2), and two other synaptic proteins, NEDD4 and Synpo, that were included to support another research project (Table 1). A list of candidate peptides corresponding to the 50 proteins was generated, and these peptides were then filtered through a set of criteria to select the optimal peptides for quantitative analysis. These criteria included minimizing the number of modifiable residues (e.g., Met, Cys, Tyr, Ser, Thr) as well as the number of flanking lysine and arginine residues to avoid potential miscleavage events. In addition, only nonredundant peptides were selected to ensure quantitation specificity. After performing this filtering, a list of 138 peptides (1–3 peptides per protein) was generated for synthesis of stable-isotope-labeled peptides. Notably, of the proteins selected for targeted PRM analysis, several contaminants were included to monitor the quality of PSD enrichment, such as GFAP, MBP, piccolo, bassoon, alpha spectrin, and various ribosomal proteins. 

### 3.7. PRM Analysis of PSD Target Proteins Revealed Quantitative Differences in Protein Expression in WT Versus Shank3B KO Mouse Brain Samples

**Figure 6:**
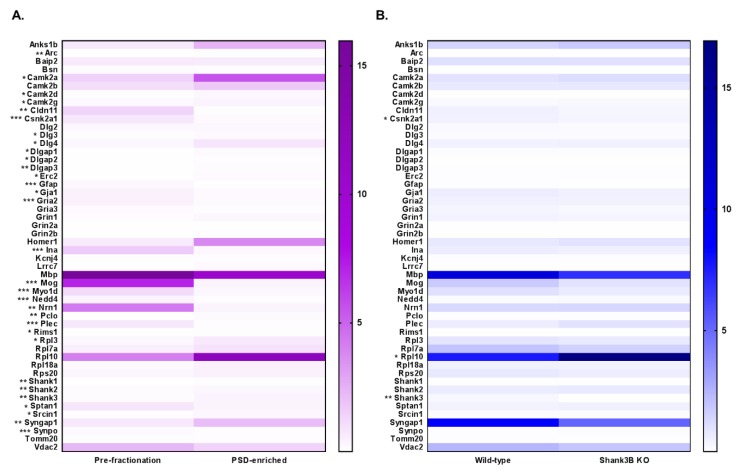
Mean-normalized protein abundance heatmap results from PRM LC–MS/MS analysis. Heatmap of analysis comparing (**A**) Pre-fractionation vs PSD-enriched samples (purple) and (**B**) WT vs Shank3B KO samples (blue). Protein accessions are listed to the left of the heatmap, and the degree of statistical significance between the two groups is designated for each protein (* = *p* < 0.05, ** = *p* < 0.01, *** = *p* < 0.005). Protein abundance is plotted as mean-normalized intensity response ratios (light/heavy), which are directly correlated with color intensity within the gradient displayed on the right of the heatmap.

To absolutely quantify PSD proteins in a more targeted approach, a PRM assay was developed for 50 known PSD and selected contaminating proteins (Table 1). Stable-isotope-labeled peptides were synthesized for 138 peptides corresponding to the 50 proteins and used as internal standards for absolute quantitation. The same sample sets that were used in the DIA assay were also used for PRM analysis. However, Sample 3 of the pre-fractionation group was injected in technical duplicate, and both were included in the quantitation. The resulting data were analyzed using Skyline software, which quantified the peak area intensities for each heavy and corresponding light peptide. The response ratios were then summed and mean-normalized for each protein (Figure S3). A protein expression heatmap was generated for each analysis (Figure 6), and a two-tailed *t*-test was performed between the two groups to determine statistical significance. 

In total, there were 31 proteins that were significantly differentially expressed (as indicated by the asterisks preceding the accession names of these proteins) in the pre-fractionation versus PSD-enriched analysis (Figure 6A). These results are displayed in Table S11. Like the DIA assay, the PSD-enriched fractions displayed significantly increased expression levels of PSD proteins, including those in the MAGUK, Shank, and GKAP families. Three out of four of the CaMKII subunits had significantly increased abundance in the PSD fractions, with CaMKIIb trending in a similar direction (p=0.059). Interestingly, AMPA receptor Gria2 displayed significantly decreased expression in the PSD-enriched fractions compared to pre-fractionation samples, which was the inverse of the results observed in the DIA analysis. However, after assessment of the peptides identified for Gria2 in the DIA analysis (26 total), it seemed that this discrepancy was largely driven by the 24 Gria2 peptides that were unique to the DIA assay. That is, the two peptides ADIAIAPLTITLVR and LTIVGDGK, which were common to both the DIA and PRM assays showed similar trends in expression in both assays. Furthermore, expression profiles of PSD-contaminating proteins including alpha spectrin, myelin-oligodendrocyte glycoprotein (Mog), GFAP, and plectin indicated significant decreases in protein expression in PSD fractions compared to pre-fractionation samples.

The second PRM analysis compared WT and KO Shank3B fractions and revealed three significantly, differentially expressed proteins, including a 12-fold decrease (p=0.005) in Shank3 protein in KO fractions (Figure 6B), a decrease that was also observed in the DIA assay. These results are displayed in Table S12. Again, a low level of Shank3 expression was still present in the KO fractions, since the three selected Shank3 target peptides were not exclusive to the Shank3B isoform. For instance, while peptides AALAVGSPGPVGGSFAR and LDPTAPVWAAK were not present in the Shank3B sequence, they were found in eight and one other Shank3 isoforms, respectively. Conversely, both Shank3B and three other isoforms contained the third Shank3 peptide, VLSIGEGGFWEGTVK, in the PRM assay (Figure S4). In addition to Shank3, Csnk2a1 (CK2) and ribosomal protein L10 (Rpl10) were found to be significantly differentially expressed in WT versus KO samples. A significant increase in Csnk2a1 (*p* = 0.017) expression in WT compared to KO fractions was observed, while the inverse was true for Rpl10 (*p* = 0.048). Although the DIA expression profiles for these proteins were trending in similar directions as in the PRM assays, the levels of differential expression seen in the DIA assays were not statistically significant. A complete list of experimental results for both PRM analyses can be found in Table S13. Collectively, these results indicated that the PRM assay can be utilized for accurate quantitation of PSD proteins in both fractionated and unfractionated samples for biological characterization.

## 4. Discussion

Collectively, these assays demonstrated the power and selectivity of targeted mass spectrometry for quantitation of PSD proteins. Performing PRM and DIA assays in parallel enabled the identification and quantitation of over 2000 proteins before and after enrichment of the PSD from mouse cortical tissue. Many of these proteins displayed similar trends in both assays, including the scaffolding protein Shank3, which had significantly decreased expression in Shank3B knockout PSD samples compared to wild-type samples. Furthermore, proteins that have routinely been identified in PSD fractions in other proteomics studies, such as PSD-95, DLGAPs, and glutamate receptors (Gria), displayed significantly increased expression in PSD fractions compared to pre-enrichment samples in both PRM and DIA assays. 

Though many of the proteins displayed similar expression profiles in both assays, there were also some discrepancies which can be attributed to differences in the number and specific peptides identified and quantified in each protein. For instance, CaMKIIa was significantly increased (*p* = 0.015) in Shank3B KO PSD samples compared to WT after DIA analysis, which identified 14 total peptides for CaMKIIa. However, PRM analysis of three peptides corresponding to CaMKIIa in the same samples resulted in a quantitative profile that was trending in a similar direction, but the expression difference was not significant with a t-test. Conversely, Csnk2a1 displayed a significant decrease (*p* = 0.017) in expression in KO versus WT samples after PRM analysis, while there was no significant difference in expression after DIA analysis of the same samples. However, the DIA analysis used five total peptides to quantify Csnk2a1, and only two out of the three PRM target peptides were identified and included in the DIA quantitation. These differences illustrate the importance of careful design, optimization, and validation of targeted assays for quantitative proteomics. In addition to mass spectrometry method development, sample selection also becomes important to determine the utility of the assays. This is one reason why two different sample sets were used for initial validation of the PSD targeted assays. 

The quantification of proteins from pre-fractionated samples and PSD-enriched samples of mouse cortical tissues was initially performed to demonstrate the selectivity and utility of these assays for different sample types. Determining the limit of detection and quantitation of these proteins allows one to assess the degree of PSD enrichment and the level of contaminating proteins, which is commonly performed using methods such as immunoblot analysis. The second comparison of PSD proteins from Shank3B KO and WT mice was selected on the basis of prior Shank3-related proteomic analyses [15,74]. The Shank3B knockout line used in our study was originally generated by homologous recombination that resulted in the disruption of the PDZ domain of Shank3B (exon 13-16) [15]. Initial proteomic characterization of this knockout line was performed in striatal synapses using immunoblot analysis, which revealed a significant decrease in protein expression of many characteristic PSD proteins in KO versus WT, including PSD-95, glutamate receptors, and CaMKIIa [15]. Interestingly, DIA analysis of mouse cortical tissue revealed inverse results to those seen in the Peça et al. study; however, this difference may be attributed to the brain region analyzed, as Shank3, but not Shank1 or Shank 2, is highly expressed in the striatum of mouse brain [15]. Another study used ion-mobility-enhanced DIA analysis to assess changes in the striatal and hippocampal proteomes of Shank3Δ11*^-/-^* knockout mice, revealing significant decreases in the expression of glutamate receptors, including Grin1, Grin2B, Gria1, and Gria2, compared to wild-type [74]. Both PRM and DIA analysis of mouse cortical tissues did not show significant differences in glutamate receptors between WT and Shank3B KO animals, which again could be due to differences in the brain regions analyzed and to differences in the knockout mouse lines used. 

In conclusion, we report on the validation and utilization of both PRM and DIA assays for quantitation of PSD proteins, which have now been demonstrated on two different sample sets. These assays provide a high-mass-accuracy, reproducible method for quantitation of PSD proteins that can be used as tools for a variety of applications in mouse or rat brain tissue. Together, the results from these analyses show promise for future studies of PSD proteomics and neurological disorders. 

## Figures and Tables

**Table 1:** List of target proteins and peptides for parallel reaction monitoring (PRM) analysis.

Protein#.	Gene Name	Protein Description	Peptide #	Peptide Sequence
1	Anks1b	Ankyrin repeat & sterile alpha motif domain-containing protein 1B	1	TLANLPWIVEPGQEAK
			2	LIFQSCDYK
			3	ILQAIQLLPK
2	Arc	Activity-regulated cytoskeleton-associated protein	4	GGPAAKPNVILQIGK
			5	TLEQLIQR
3	Baiap2	Brain-specific angiogenesis inhibitor 1-associated protein 2	6	EGDLITLLVPEAR
			7	AFHNELLTQLEQK
			8	AIFSHAAGDNSTLLSFK
4	Bsn	Protein bassoon	9	ATAEFSTQTPSLTPSSDIPR
			10	HGGGSGGPDLVPYQPQHGPGLNAPQGLASLR
			11	ATSVPGPTQATAPPEVGR
5	Camk2a	Calcium/calmodulin-dependent protein kinase type II subunit alpha	12	FTEEYQLFEELGK
			13	VLAGQEYAAK
			14	ITQYLDAGGIPR
6	Camk2b	Calcium/calmodulin-dependent protein kinase type II subunit beta	15	TTEQLIEAVNNGDFEAYAK
			16	GSLPPAALEPQTTVIHNPVDGIK
			17	ESSDSTNTTIEDEDAK
7	Camk2d	Calcium/calmodulin-dependent protein kinase type II subunit delta	18	FTDEYQLFEELGK
			19	IPTGQEYAAK
8	Camk2g	Calcium/calmodulin-dependent protein kinase type II subunit gamma	20	FYFENLLSK
			21	ITEQLIEAINNGDFEAYTK
			22	FTDDYQLFEELGK
9	Cldn11	Claudin-11	23	FYYSSGSSSPTHAK
10	Csnk2a1	CK2	24	GGPNIITLADIVKDPVSR
			25	TPALVFEHVNNTDFK
			26	LIDWGLAEFYHPGQEYNVR
11	Dlg2	Disks large homolog 2	27	DSGLPSQGLSFK
			28	GQEDLILSYEPVTR
			29	FIEAGQYNDNLYGTSVQSVR
12	Dlg3	Disks large homolog 3	30	VNEVDVSEVVHSR
			31	ILSVNGVNLR
			32	LLAVNNTNLQDVR
13	Dlg4	PSD-95	33	NAGQTVTIIAQYKPEEYSR
			34	EVTHSAAVEALK
			35	IIPGGAAAQDGR
14	Dlgap1	Disks large-associated protein 1	36	AVSEVSINR
			37	FQSVGVQVEEEK
			38	SLDSLDPAGLLTSPK
15	Dlgap2	Disks large-associated protein 2	39	TQGLFSYR
			40	CSSIGVQDSEFPDHQPYPR
			41	TSPTVALRPEPLLK
16	Dlgap3	Disks large-associated protein 3	42	EAEDYELPEEILEK
			43	FLELQQLK
			44	GPAGPGPGPGSGAAPEAR
17	Erc2	ERC protein 2	45	DLNHLLQQESGNR
			46	VNALQAELTEK
			47	IAELESLTLR
18	Gfap	Glial fibrillary acidic protein	48	ALAAELNQLR
			49	ITIPVQTFSNLQIR
			50	LADVYQAELR
19	Gja1	Gap junction alpha-1 protein	51	SDPYHATTGPLSPSK
20	Gria2	Glutamate receptor 2	52	LTIVGDGK
			53	ADIAIAPLTITLVR
			54	GADQEYSAFR
21	Gria3	Glutamate receptor 3	55	GSALGNAVNLAVLK
			56	NTQNFKPAPATNTQNYATYR
			57	ADIAVAPLTITLVR
22	Grin1	Glutamate receptor ionotropic, NMDA 1	58	VIILSASEDDAATVYR
			59	HNYESAAEAIQAVR
			60	IPVLGLTTR
23	Grin2a	Glutamate receptor ionotropic, NMDA 2A	61	FSYIPEAK
			62	GVEDALVSLK
			63	YLPEEVAHSDISETSSR
24	Grin2b	Glutamate receptor ionotropic, NMDA 2B	64	FQRPNDFSPPFR
			65	SDVSDISTHTVTYGNIEGNAAK
25	Homer1	Homer1	66	LTAALLESTANVK
			67	HAVTVSYFYDSTR
			68	ANTVYGLGFSSEHHLSK
26	Ina	Alpha-internexin	69	ALEAELAALR
			70	FANLNEQAAR
			71	HSAEVAGYQDSIGQLESDLR
27	Kcnj4	Inward rectifier potassium channel 4	72	FEPVVFEEK
			73	SSYLASEILWGHR
			74	TYEVAGTPCCSAR
28	Lrrc7	Leucine-rich repeat-containing protein 7	75	VLNLSDNR
			76	ALIPLQTEAHPETK
			77	IVGVPLELEQSTHR
29	Mbp	Myelin basic protein	78	DTGILDSIGR
			79	TPPPSQGK
			80	TQDENPVVHFFK
30	Mog	Myelin-oligodendrocyte glycoprotein	81	ALVGDEAELPCR
			82	DQDAEQAPEYR
			83	FSDEGGYTCFFR
31	Myo1d	Unconventional myosin-1d	84	VVSVIAELLSTK
			85	HQVEYLGLLENVR
			86	IGELVGVLVNHFK
32	Nedd4	E3 ubiquitin-protein ligase NEDD4	87	EWFFLISK
			88	LLDGFFIRPFYK
			89	LLQFVTGTSR
33	Nrn1	Neuritin	90	FSTFSGSITGPLYTHR
			91	GFSDCLLK
34	Pclo	Protein piccolo	92	NYVLIDDIGDITK
			93	AQEAEALDVSFGHSSSSAR
			94	AAAGPLPPISADTR
35	Plec	Plectin	95	DSQDAGGFGPEDR
			96	IISLETYNLFR
			97	LGFHLPLEVAYQR
36	Rims1	Regulating synaptic membrane exocytosis protein 1	98	ATTLTVPEQQR
			99	ESGALLGLK
			100	ETSPISSHPVTWQPSK
37	Rpl3	60S ribosomal protein L3	101	VACIGAWHPAR
			102	IGQGYLIKDGK
			103	NNASTDYDLSDK
38	Rpl7a	60S ribosomal protein L7a	104	NFGIGQDIQPK
			105	LKVPPAINQFTQALDR
			106	AGVNTVTTLVENK
39	Rpl10	60S ribosomal protein L10	107	VHIGQVIMSIR
40	Rpl18a	60S ribosomal protein L18a	108	IFAPNHVVAK
			109	VKNFGIWLR
			110	DLTTAGAVTQCYR
41	Rps20	40S ribosomal protein S20	111	DTGKTPVEPEVAIHR
			112	VCADLIR
			113	LIDLHSPSEIVK
42	Shank1	SH3 and multiple ankyrin repeat domains protein 1	114	ALTASPPAAR
			115	LESGGSSGGYGAYAAGSR
			116	GSSTEDGPGVPPPSPR
43	Shank2	SH3 and multiple ankyrin repeat domains protein 2	117	AASVPALADLVK
			118	LLDPSSPLALALSAR
			119	IFLSGITEEER
44	Shank3	SH3 and multiple ankyrin repeat domains protein 3	120	AALAVGSPGPVGGSFAR
			121	LDPTAPVWAAK
			122	VLSIGEGGFWEGTVK
45	Sptan1	Spectrin alpha chain, non-erythrocytic 1	123	ELPTAFDYVEFTR
			124	SSLSSAQADFNQLAELDR
			125	HQAFEAELSANQSR
46	Srcin1	SRC kinase signaling inhibitor 1	126	GEGLYADPYGLLHEGR
			127	AGAGGPLYGDGYGFR
			128	LLEETQAELLK
47	Syngap1	Ras GTPase-activating protein SynGAP	129	AGYVGLVTVPVATLAGR
			130	GGEPPGDTFAPFHGYSK
			131	SASGDTVFWGEHFEFNNLPAVR
48	Synpo	Synaptopodin	132	YVIESSGHAELAR
			133	AASPAKPSSLDLVPNLPR
			134	VASEEEEVPLVVYLK
49	Tomm20	Mitochondrial import receptor subunit TOM20	135	LPDLKDAEAVQK
50	Vdac2	Voltage-dependent anion-selective channel protein 2	136	GFGFGLVK
			137	YQLDPTASISAK
			138	WCEYGLTFTEK

^1^ List of proteins and corresponding tryptic peptides targeted in the PSD PRM assay. Stable-isotope-labeled (SIL) peptides were synthesized with the label incorporated in the C-terminal arginine (R) or lysine (K) residue of each peptide.

## Supplementary Materials

The following are available online at https://www.mdpi.com/2227-7382/7/2/12/s1, Figures S1–S4; Tables S1–S13.
