# Supplementary material for: Development of Targeted Mass Spectrometry-Based Approaches for Quantitation of Proteins Enriched in the Postsynaptic Density (PSD)
Source: Proteomes. 2019 Apr 2;7(2):12. doi: 10.3390/proteomes7020012 (PMC6630806; doi:10.3390/proteomes7020012)
Supplement: Supplementary file 1 [file proteomes-07-00012-s001.zip › Supplementary Figures.pptx]

## Slide 1
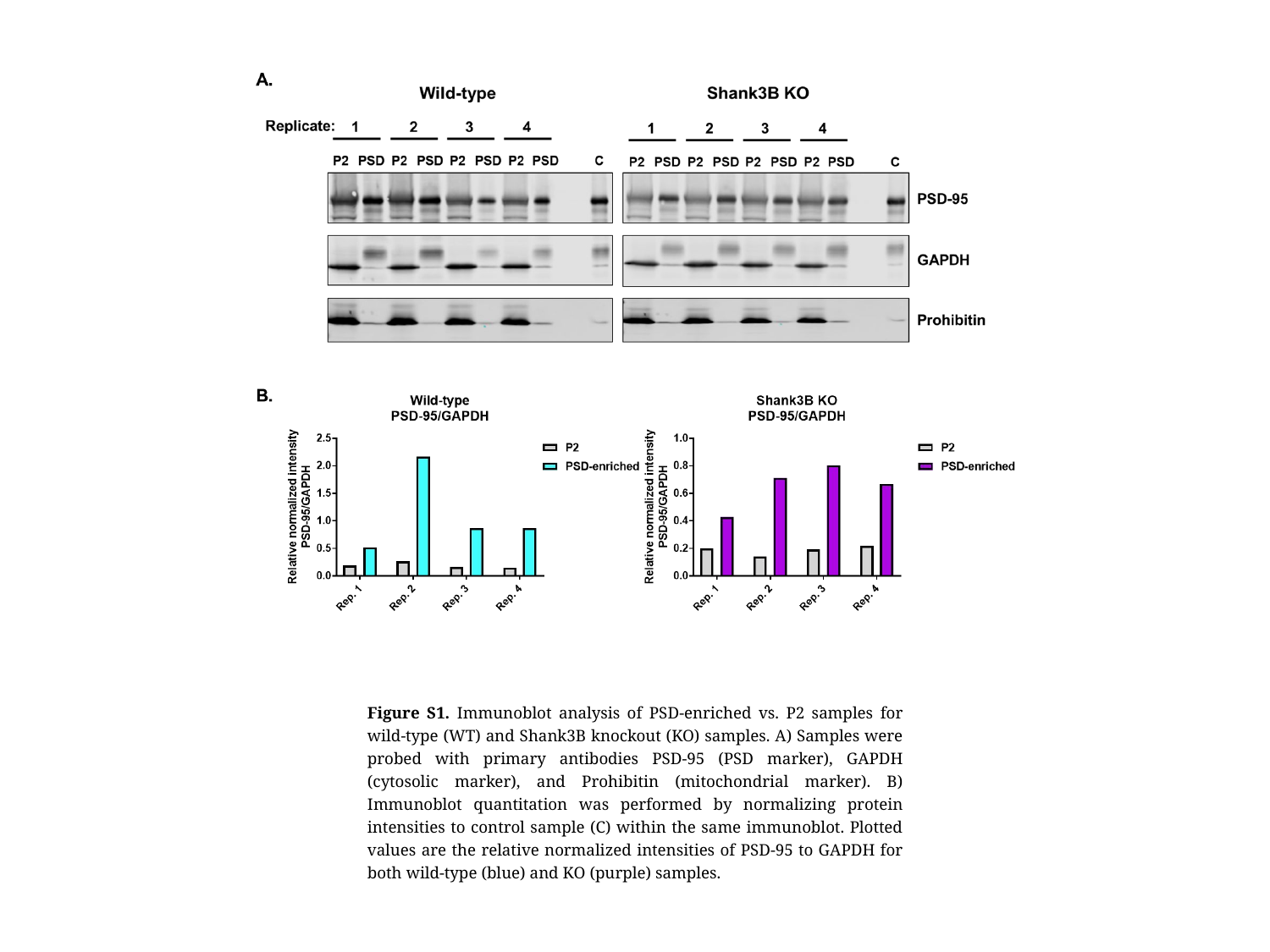

Figure S1. Immunoblot analysis of PSD-enriched vs. P2 samples for wild-type (WT) and Shank3B knockout (KO) samples. A) Samples were probed with primary antibodies PSD-95 (PSD marker), GAPDH (cytosolic marker), and Prohibitin (mitochondrial marker). B) Immunoblot quantitation was performed by normalizing protein intensities to control sample (C) within the same immunoblot. Plotted values are the relative normalized intensities of PSD-95 to GAPDH for both wild-type (blue) and KO (purple) samples.

## Slide 2
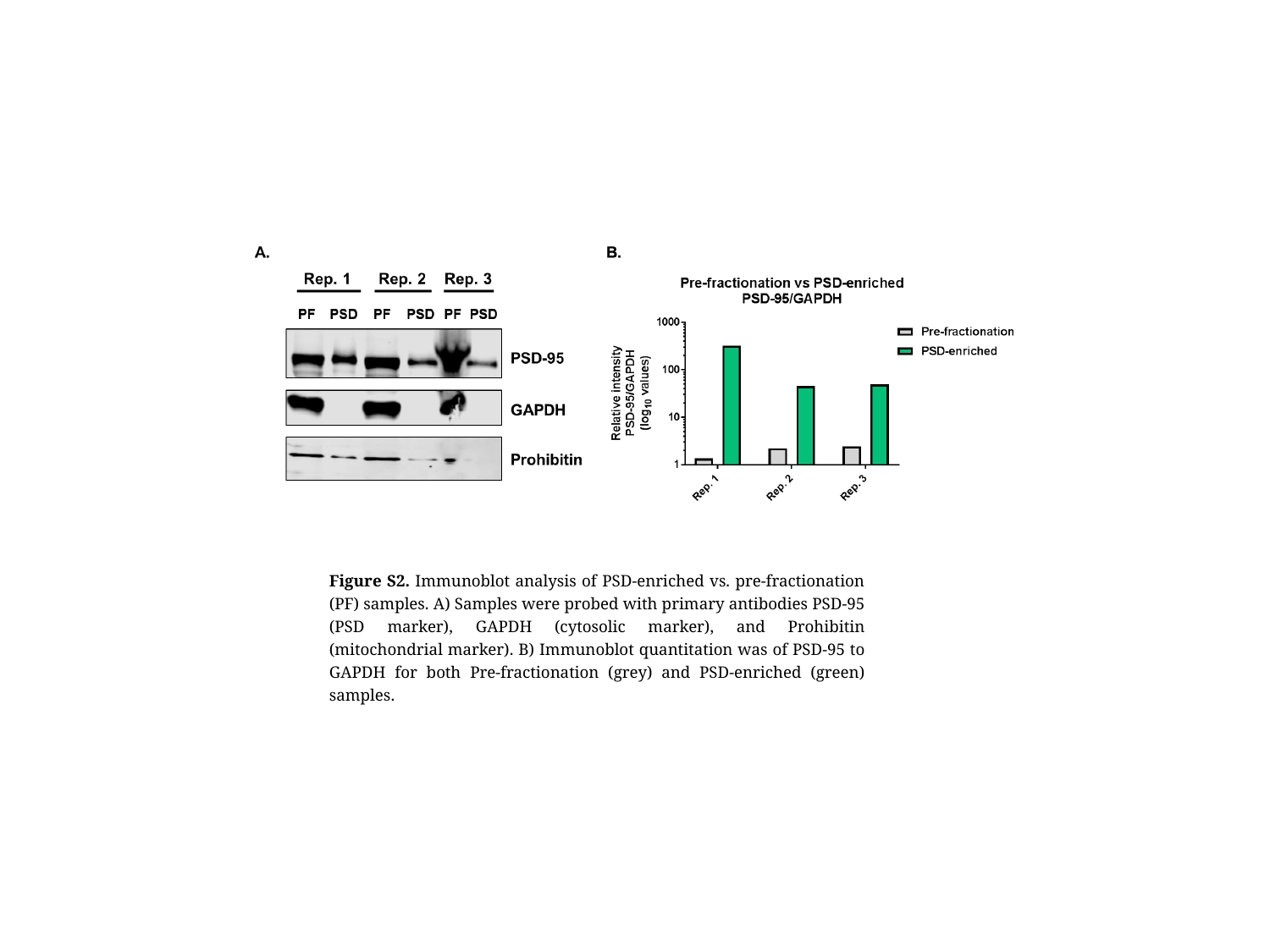

Figure S2. Immunoblot analysis of PSD-enriched vs. pre-fractionation (PF) samples. A) Samples were probed with primary antibodies PSD-95 (PSD marker), GAPDH (cytosolic marker), and Prohibitin (mitochondrial marker). B) Immunoblot quantitation was of PSD-95 to GAPDH for both Pre-fractionation (grey) and PSD-enriched (green) samples.

## Slide 3
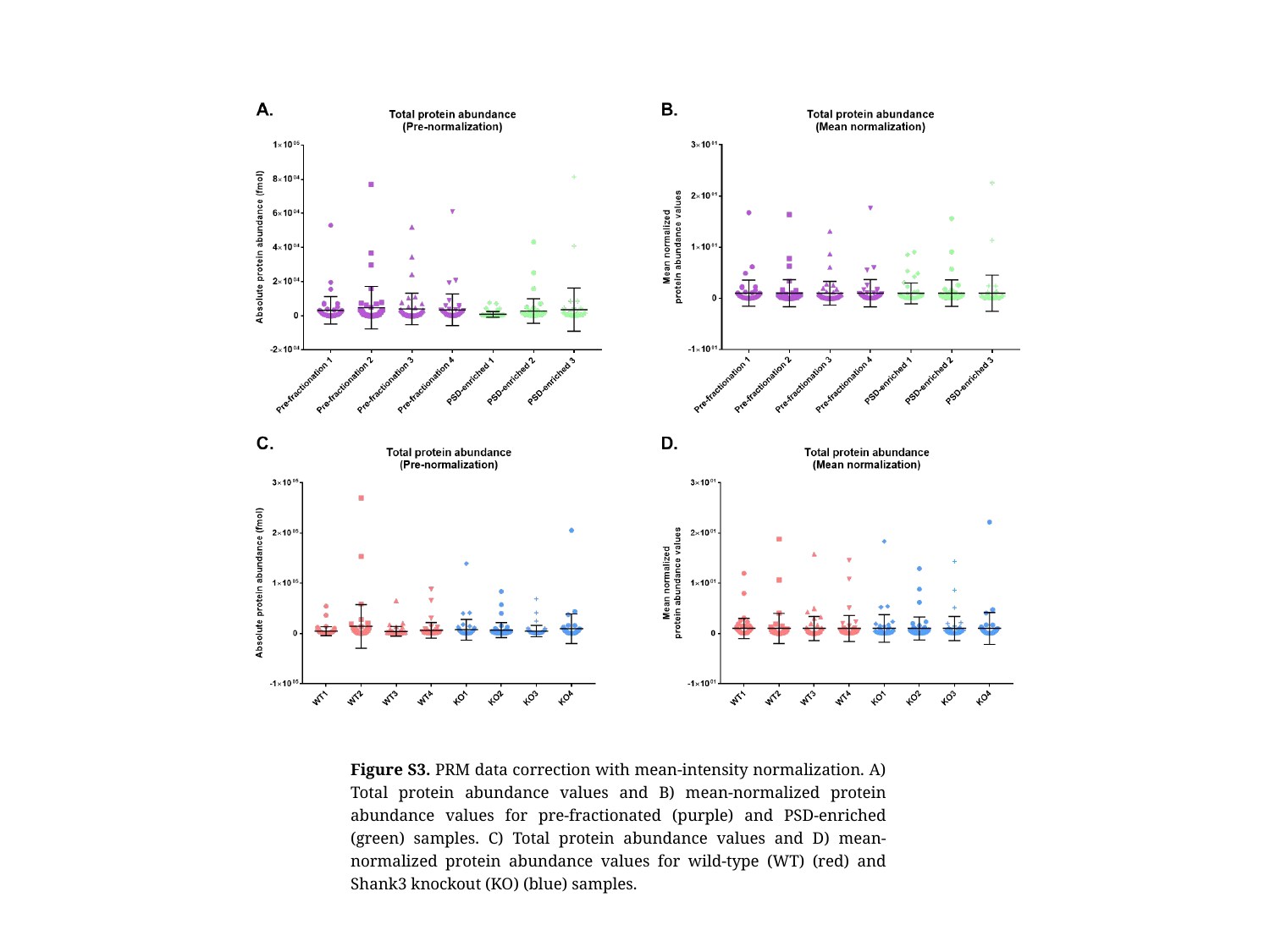

Figure S3. PRM data correction with mean-intensity normalization. A) Total protein abundance values and B) mean-normalized protein abundance values for pre-fractionated (purple) and PSD-enriched (green) samples. C) Total protein abundance values and D) mean-normalized protein abundance values for wild-type (WT) (red) and Shank3 knockout (KO) (blue) samples.

## Slide 4
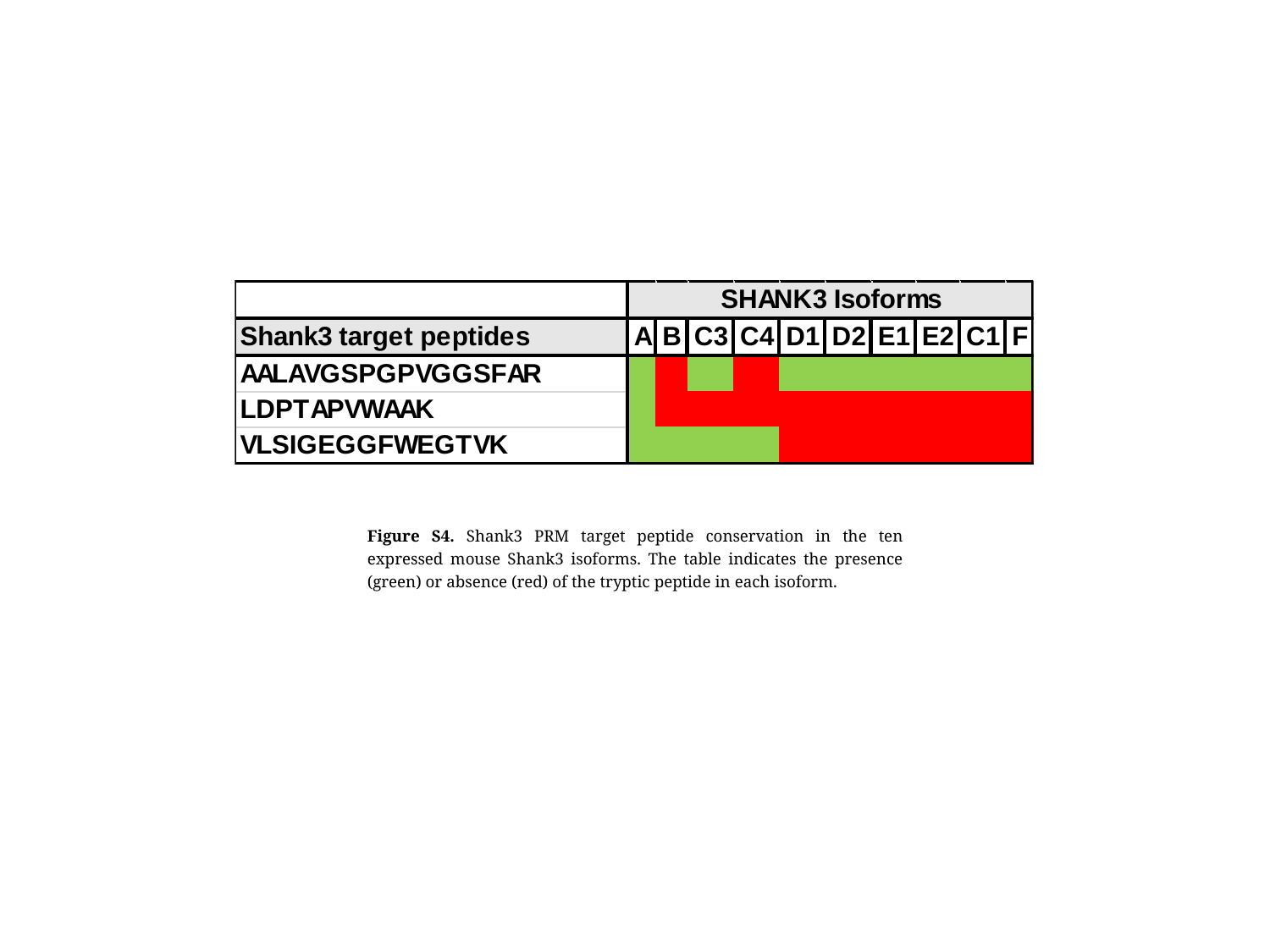

Figure S4. Shank3 PRM target peptide conservation in the ten expressed mouse Shank3 isoforms. The table indicates the presence (green) or absence (red) of the tryptic peptide in each isoform.
